# Development of a High-Throughput Flow Cytometry Assay to Monitor Defective Trafficking and Rescue of Long QT2 Mutant hERG Channels

**DOI:** 10.3389/fphys.2018.00397

**Published:** 2018-04-19

**Authors:** Scott A. Kanner, Ananya Jain, Henry M. Colecraft

**Affiliations:** ^1^Doctoral Program in Neurobiology and Behavior, Columbia University College of Physicians and Surgeons, New York, NY, United States; ^2^Department of Physiology and Cellular Biophysics, Columbia University College of Physicians and Surgeons, New York, NY, United States; ^3^Department of Pharmacology, Columbia University College of Physicians and Surgeons, New York, NY, United States

**Keywords:** long QT syndrome type 2, HERG channels (Kv11), ion channel trafficking, flow cytometry, cardiac arrhythmias

## Abstract

Long QT Syndrome (LQTS) is an acquired or inherited disorder characterized by prolonged QT interval, exertion-triggered arrhythmias, and sudden cardiac death. One of the most prevalent hereditary LQTS subtypes, LQT2, results from loss-of-function mutations in the hERG channel, which conducts *I*_Kr_, the rapid component of the delayed rectifier K^+^ current, critical for cardiac repolarization. The majority of LQT2 mutations result in Class 2 deficits characterized by impaired maturation and trafficking of hERG channels. Here, we have developed a high-throughput flow cytometric assay to analyze the surface and total expression of wild-type (WT) and mutant hERG channels with single-cell resolution. To test our method, we focused on 16 LQT2 mutations in the hERG Per-Arnt-Sim (PAS) domain that were previously studied via a widely used biochemical approach that compares levels of 135-kDa immature and 155-kDa fully glycosylated hERG protein to infer surface expression. We confirmed that LQT2 mutants expressed in HEK293 cells displayed a decreased surface density compared to WT hERG, and were differentially rescued by low temperature. However, we also uncovered some notable differences from the findings obtained via the biochemical approach. In particular, three mutations (N33T, R56Q, and A57P) with apparent WT-like hERG glycosylation patterns displayed up to 50% decreased surface expression. Furthermore, despite WT-like levels of complex glycosylation, these mutants have impaired forward trafficking, and exhibit varying half-lives at the cell surface. The results highlight utility of the surface labeling/flow cytometry approach to quantitatively assess trafficking deficiencies associated with LQT2 mutations, to discern underlying mechanisms, and to report on interventions that rescue deficits in hERG surface expression.

## Introduction

Long QT Syndrome (LQTS) is an inherited or acquired disorder characterized by delayed cardiac action potential repolarization, which predisposes to polymorphic ventricular tachycardias (torsade de pointes), syncope, and sudden cardiac death (SCD) (Moss and Kass, 2005; Bohnen et al., 2016). Congenital LQTS occurs in approximately 1 in 2000 live births (Schwartz et al., 2009), and accounts for a significant portion of ∼400,000 cases of SCD in the United States each year (Tester and Ackerman, 2009; George, 2013). Loss-of-function mutations in several genes have been linked to LQTS (LQT1-LQT13), with around 70% occurring in genes encoding pore-forming subunits for the primary repolarizing K^+^ currents in ventricular cardiomyocytes – KCNQ1 (LQT1) and hERG (LQT2) (Bohnen et al., 2016).

The hERG potassium channels assemble as a tetramer of four Kv11.1 α_1_ pore-forming subunits, and conduct *I*_Kr_, the rapid component of the delayed rectifier K^+^ current (Trudeau et al., 1995). *I*_Kr_ is critical for proper cardiac repolarization, as well as the suppression of arrhythmic events caused by premature stimuli (Sanguinetti et al., 1995; Vandenberg et al., 2012). Over 500 LQT2 mutations in hERG have been described to date, with ∼40% consisting of non-sense mutations and ∼60% being missense mutations (Schwartz et al., 2009; Smith et al., 2016). Four classes of mutations have been described: Class 1 mutations affect channel synthesis or translation; Class 2 mutations affect channel trafficking and intracellular transportation; Class 3 mutations alter channel gating; and Class 4 affect ion permeability (Smith et al., 2016). It has become apparent that the vast majority (∼88%) of LQT2 mutations are Class 2 type, featuring compromised channel trafficking to the plasma membrane (Anderson et al., 2006, 2014; Smith et al., 2016). Understanding the mechanisms regulating hERG trafficking and how these may be dysregulated in disease is important for molecular insights into the pathophysiology of LQT2.

**FIGURE 1:**
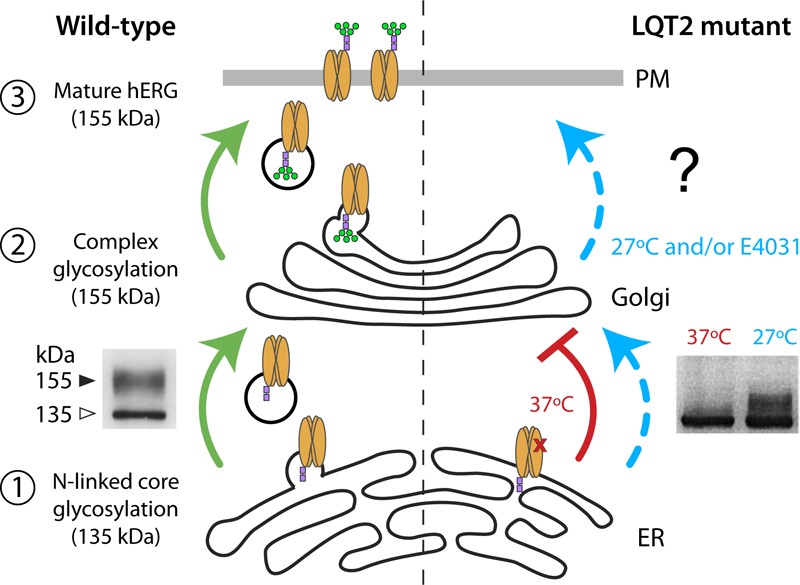
hERG maturation, glycosylation, and surface trafficking. (Left) Forward trafficking of WT hERG (green) is represented. WT hERG undergoes assembly and post-translational N-linked core glycosylation (purple squares) in the endoplasmic reticulum (step 1) and complex glycosylation (green circles) in the Golgi (step 2). Functional channels on the cell surface contain the fully glycosylated 155-kDa mature form of hERG (step 3). Immunoblot (inset, Left) adapted from Gong et al. (2002) represents core glycosylated (135-kDa) and complex glycosylated (155-kDa) hERG bands. (Right) Correctable LQT2 mutant (i.e., Class 2 trafficking defect) is represented at 37°C (red) and incubation with low temperature/chemical chaperone (blue). Immunoblot (inset, Right) adapted from Zhou et al. (1999) demonstrates temperature-dependent rescue of complex glycosylation.

The hERG channels undergo several levels of post-translational processing and maturation before the functional channel reaches the cell surface (**Figure 1**). They are synthesized in the endoplasmic reticulum (ER), which provides an environment for optimum folding and assembly. In the ER, the 132-kDa protein undergoes N-linked core glycosylation of the protein, generating a 135-kDa immature protein (Zhou et al., 1998b; Petrecca et al., 1999; Gong et al., 2002) (**Figure 1**, left – step 1). From the ER, hERG that is properly folded and assembled is exported via COPII vesicles (Delisle et al., 2009) to the Golgi where it is matured through N-linked complex glycosylation to generate a 155-kDa protein that is biochemically distinguishable from the immature form (**Figure 1**, left – step 2). Most studies investigating hERG trafficking defects in inherited or acquired LQT2 have taken advantage of these biochemical signatures, utilizing immunoblot assays to distinguish relative expression of 135-kDa immature and 155-kDa mature bands and, thereby, infer surface expression (**Figure 1**, left – step 3) (Zhou et al., 1998b, 1999; Gong et al., 2002, 2005; Guo et al., 2009; Dennis et al., 2011; Apaja et al., 2013; Ke et al., 2013).

The conclusion that a majority of LQT2 mutations are of the Class 2 type (i.e., trafficking-deficient) come from observations that there is either a relative or a complete loss of the 155-kDa mature hERG protein in most of these instances (Anderson et al., 2006, 2014; Ke et al., 2013). An important feature of many trafficking-deficient LQT2 mutants is that they are not irretrievably lost, but can be rescued by incubation at low temperature or with chemical chaperones such as the Kv11.1 channel blocker, E-4031 (Zhou et al., 1999; Ficker et al., 2002; Anderson et al., 2006). Such correction typically results in the re-emergence of the mature 155-kDa band, thereby providing a signature that can be monitored to evaluate rescue efficacy (**Figure 1**, right).

Despite the evident efficacy of the biochemical assay to probe hERG protein trafficking, there are some potential limitations to this approach. First, it does not provide a direct measure of surface channels. This is pertinent giving findings that glycosylation may not be absolutely required for surface trafficking (Gong et al., 2002), and the possibility that some fully glycosylated channels may still be compromised in their ability to reach the cell surface (Dennis et al., 2011; Smith et al., 2011). Second, the method may lack subcellular discrimination since glycosylated channels could potentially reside in the Golgi as well as post-Golgi compartments including the cell surface and endosomes. Finally, the biochemical approach is relatively labor-intensive which limits opportunities for rapid high throughput screening strategies to identify novel trafficking correctors as potential therapeutics. Here, we sought to develop an optical high-throughput assay to monitor surface and total hERG protein expression that would be useful to discern mechanisms underlying LQT2 trafficking deficiencies and also amenable as an assay for identifying new hERG protein trafficking correctors.

## Results

### Design and Implementation of a Flow Cytometric Assay to Analyze WT and Mutant hERG Surface Expression

**FIGURE 2:**
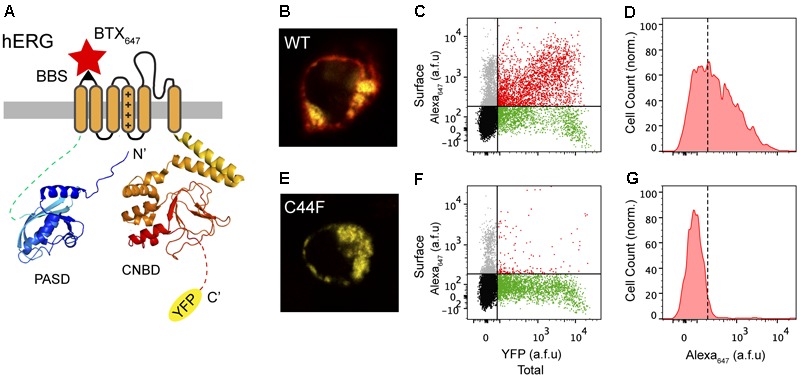
Surface labeling of hERG channels. **(A)** Cartoon of BBS-hERG-YFP subunit. PASD (blue) and CNBD (red) structures adapted from Wang and MacKinnon (2017) (PDB: 5VA2). The bungarotoxin binding site (BBS) epitope (S1-S2) allows for selective labeling of surface hERG channels while YFP signal represents total hERG protein expression. **(B)** Confocal image of a live cell expressing BBS-hERG-YFP and stained with BTX_647_. **(C)** Flow cytometry dot plot showing surface (BTX_647_ fluorescence) and total (YFP fluorescence) hERG expression in cells expressing BBS-hERG-YFP. Vertical and horizontal lines represent thresholds for YFP and BTX_647_-positive cells, respectively, based on the analyses of single color controls. Represented are YFP-positive cells with BTX_647_ signal above (red dots) or below threshold (green dots); BTX_647_-positive cells with YFP signal below threshold (gray dots); and untransfected cells (black dots). **(D)** Histogram of BTX_647_ fluorescence in cells expressing WT BBS-hERG-YFP, generated from population of YFP-positive cells. Dotted line is threshold value for BTX_647_ signal. **(E–G)** Data for cells expressing LQT2 mutant BBS-hERG_C44F_-YFP channels; same format as **B–D**.

Previous studies from our laboratory and others have shown the utility of a 13-residue high-affinity bungarotoxin binding site (BBS) introduced as an extracellular epitope tag to faithfully label surface population of distinct ion channels and membrane proteins (Sekine-Aizawa and Huganir, 2004; Wilkins et al., 2008; Yang et al., 2010; Aromolaran et al., 2014; Cassidy et al., 2014). Building on previous work that utilized an extracellular HA epitope for surface detection of hERG (Ficker et al., 2003; Wible et al., 2005), we introduced the BBS tag into the extracellular S1-S2 loop of hERG to enable efficient detection of surface channels in non-permeabilized cells with Alexa Fluor 647-conjugated bungarotoxin (BTX_647_) (**Figure 2A**). We also fused YFP to the C-terminus of hERG to enable simultaneous fluorescence detection of total hERG expression (**Figure 2A**). Human embryonic kidney (HEK293) cells transiently transfected with wild-type (WT) BBS-hERG-YFP displayed robust fluorescence signals for total (yellow; YFP) and surface (red; BTX_647_) channel pools when imaged by confocal microscopy (**Figure 2B**). We used flow cytometry to quantify total and surface BBS-hERG-YFP channel pools in an unbiased and high-throughput manner, all with single cell resolution (**Figure 2C**). Consistent with the confocal microscopy results, cells expressing WT BBS-hERG-YFP (i.e., YFP-positive cells) displayed robust surface expression, with red fluorescence signals up to a 100-fold higher than a threshold level established with single color controls (**Figures 2C,D**).

As an initial test of the robustness of this assay to report on trafficking-deficient LQT2 mutants, we examined the impact of introducing an LQT2-causing point mutation in hERG, C44F, which is known to be trafficking-deficient as it is not post-translationally processed to the 155-kDa mature form of the protein (Lupoglazoff et al., 2001; Anderson et al., 2014). Consistent with this view, mutant BBS-hERG_C44F_-YFP displayed no surface BTX_647_ red fluorescence in YFP-positive cells (**Figures 2E–G**).

### Surface Labeling Assay Reveals Distinct Subtypes of LQT2-Causing Mutations in the PAS Domain

**FIGURE 3:**
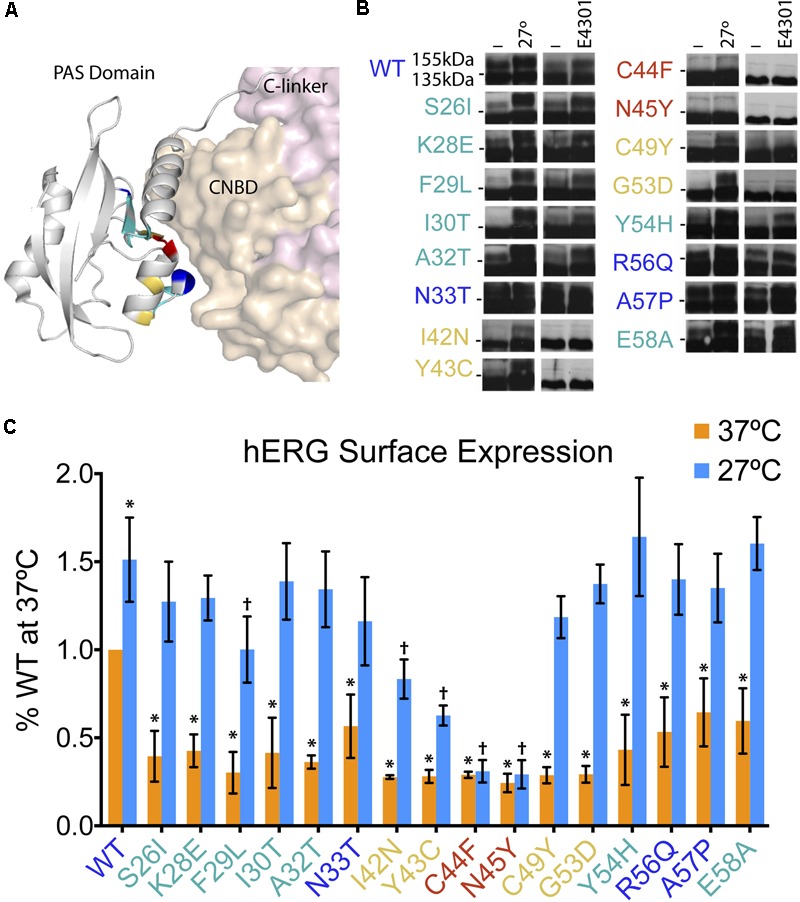
Low temperature rescue of defective surface trafficking in 16 LQT2 hERG channels with mutations in the PAS domain. **(A)** Structure of the hERG PAS domain in complex with CNBD, adapted from Wang and MacKinnon (2017) (PDB: 5VA2). Positions of 16 LQT2 mutations highlighted according to their reported impact on hERG trafficking as assessed by the prevalence of the 155-kDa mature protein and the rescue of this band by low temperature and/or E4031–WT glycosylation (blue), uncorrectable (red), temperature correctable only (yellow), and temperature and E4031 correctable (teal). **(B)** Western blot analyses of PAS domain LQT2 mutants reproduced from Anderson et al. (2014) (with permission from Nature Communications). Horizontal dashes at the sides of the blots represent 140 kDa. **(C)** Quantification of surface hERG channels (Alexa_647_ fluorescence) from flow cytometry experiments (*n* = 8878–30497 cells; *N* = 4) for WT and LQT2 mutant hERG channels at 37 and 27°C. Data are normalized to WT hERG surface expression at 37°C. ^∗^*p* < 0.02 versus WT 37°C, †*p* < 0.02 versus WT 27°C, two-way ANOVA followed by Dunnett’s test.

The hERG1a protein contains two major intracellular domains – the N-terminal Per-Arnt-Sim (PAS) domain and C-terminal cyclic nucleotide binding domain (CNBD) – which interact and require proper folding for effective channel trafficking and gating (Muskett et al., 2011; Gianulis et al., 2013; Ng et al., 2014; Wang and MacKinnon, 2017) (**Figure 3A**). In a recent comprehensive study, Anderson et al. (2014) conducted a large-scale analyses of hERG channel mutations to better understand and characterize trafficking properties of the channel resulting from mutations in distinct domains. They expressed mutant hERG channels in a heterologous expression system, under two corrective conditions – incubation at decreased temperature (27°C) or with the drug E-4031, a potassium channel blocker and pharmacological chaperone. Utilizing the immunoblot assay, they confirmed the predominance of impaired trafficking as the mechanism underlying loss-of-function of LQT2 mutant channels, and further demonstrated five distinct subclasses of mutations: (1) WT-like, (2) correctable by low temperature alone, (3) corrected by E4031 alone, (4) corrected by both low temperature and E4031, and (5) uncorrectable by either low temperature or E4031.

The comprehensive study by Anderson et al. (2014), in combination with previous studies looking at PAS domain mutations (Gianulis and Trudeau, 2011; Harley et al., 2012; Ke et al., 2013; Perry et al., 2016), provided a valuable resource and opportunity to test the robustness of the flow cytometry approach and its potential utility in providing mechanistic information beyond that provided by the biochemical method. To accomplish this, we focused on 16 mutations clustered in the PAS domain that based on the relative prevalence of 155-kDa fully glycosylated band were previously classified as: (1) WT-like (N33T, R56Q, A57P; blue), (2) uncorrectable (C44F, N45Y; red), (3) temperature correctable only (I42N, Y43C, C49Y, G53D; yellow), and (4) both low temperature and E4031 correctable (S26I, K28E, F29L, I30T, A32T, Y54H, E58A; teal) (**Figure 3B**; Western blots reproduced from Anderson et al., 2014).

We expressed WT and mutant BBS- and YFP-tagged hERG channels in HEK293 cells, under both 37°C and low temperature (27°C) conditions. Quantification of surface intensity from four independent experiments are shown, normalized to WT surface expression at 37°C (**Figure 3C**). Reassuringly, comparison of the surface density data to the immunoblotting study shows areas of concordance. First, we observed that all the LQT2 mutant channels displayed significant deficits in surface density compared to WT when cells were incubated at 37°C. Second, incubation at 27°C resulted in rescued surface density for all mutants except C44F and N45Y. However, there were some notable differences from the previous study. We observed a significant reduction in surface density of N33T, R56Q, and A57P channels that were previously classified as WT-like based on their glycosylation pattern at 37°C (**Figure 3C**). Moreover, the low temperature rescue of I42N and Y43C channels was only partial, which was not evident by the biochemical assay (**Figures 3B,C**) (Anderson et al., 2014).

Overall, these results validate the utility of the flow cytometry approach to quantify surface expression of WT and LQT2 hERG channels in a robust and sensitive manner, and to quantitatively evaluate correction of trafficking deficiencies.

### LQT2 Mutations With WT-Like Glycosylation Patterns Demonstrate Reduced Surface Expression, Impaired Forward Trafficking, and Distinct Half-Lives at the Cell Surface

**FIGURE 4:**
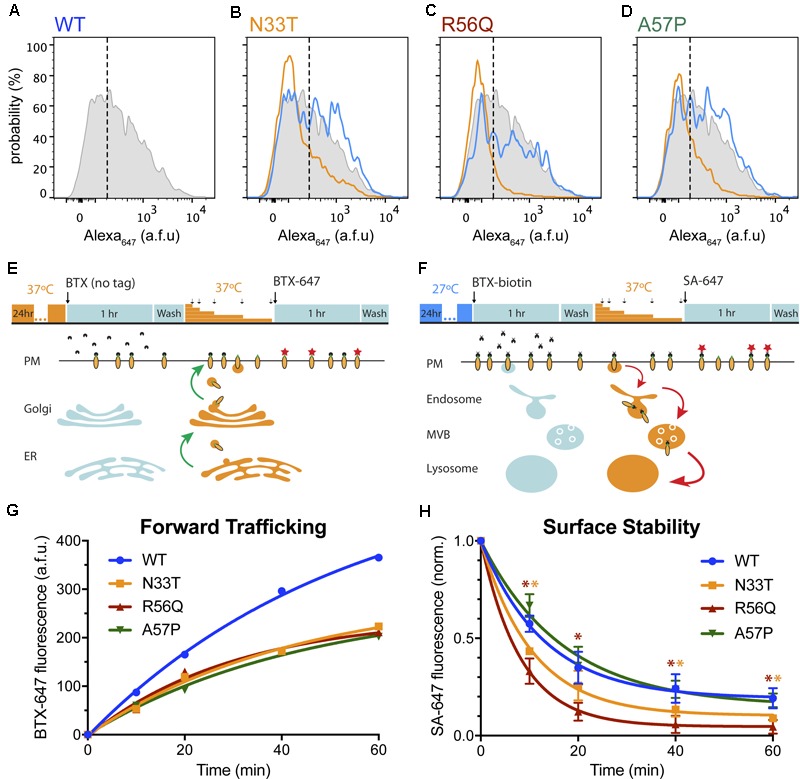
LQT2 hERG channels with WT glycosylation patterns show reduced surface expression. **(A–D)** Representative histograms representing surface intensity from HEK293 cells expressing BBS- and YFP-tagged **(A)** WT HERG (gray) at 37°C, and LQT2 mutants **(B)** N33T, **(C)** R56Q, and **(D)** A57P incubated at 37°C (orange) and 27°C (blue). **(E)** Schematic adapted from Kanner et al. (2017), showing optical pulse-chase assay for measuring BBS-hERG-YFP forward trafficking. Cells expressing BBS-hERG-YFP were incubated at 37°C for 24 h prior to the experiment. BBS-tag on channels initially at the cell surface was blocked by incubation with untagged α-bungarotoxin (BTX) at 4°C. Cells were washed and placed back at 37°C for varying time intervals (10, 20, 40, 60 min) to resume delivery of new channels to the surface membrane. Newly delivered channels were labeled with Alexa Fluor 647 conjugated BTX (BTX_647_) at 4°C and analyzed using flow cytometry. **(F)** Time evolution of BBS-hERG-YFP delivery to the surface for WT (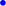, *n* = 7312–8016 cells; *N* = 2), N33T (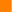, *n* = 6886–7533 cells; *N* = 2), R56Q (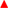, *n* = 7756–9248 cells; *N* = 2), A57P (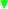, *n* = 9159–10139 cells; *N* = 2). Smooth curves are fits of an exponential growth function to the data: _y=A(1-e^-t/τ^)_. For WT (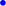), *A* = 560.7 ± 47.5, τ = 53.6 ± 9.5 min; N33T (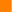), *A* = 293.1 ± 42.2, τ = 42.2 ± 12.3 min; R56Q (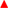), *A* = 248.6 ± 35.5, τ = 30.4 ± 10.4 min; A57P (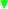), *A* = 276.2 ± 40.0, τ = 44.5 ± 12.6 min. **(G)** Schematic adapted from Kanner et al. (2017), showing optical assay for measuring BBS-hERG-YFP stability at the cell surface. Cells expressing BBS-hERG-YFP were incubated at 27°C for 24 h prior to the experiment. Channels initially at the cell surface were labeled with biotin-conjugated BTX (BTX-biotin) at 4°C. Cells were washed and incubated at 37°C for varying time intervals (10, 20, 40, 60 min) to allow for internalization of surface channels. The remaining surface channels were labeled with Alexa Fluor 647-conjugated streptavidin (SA-647) at 4°C. **(H)** Time evolution of loss of surface BBS-hERG-YFP channels WT (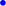, *n* = 8184–10128 cells; *N* = 3), N33T (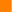, *n* = 8499–10175 cells; *N* = 3), R56Q (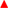, *n* = 9460–12338 cells; *N* = 3), A57P (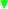, *n* = 7123–8911 cells; *N* = 3). Smooth curves are fits of an exponential decay function to the data: _y=(1-A)e^-t/τ^+A._ For WT (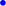), *A* = 0.19 ± 0.02, τ = 12.9 ± 0.9 min; N33T (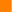), *A* = 0.10 ± 0.02, τ = 10.6 ± 0.8 min; R56Q (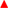), *A* = 0.05 ± 0.004, τ = 8.2 ± 0.2 min; A57P (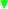), *A* = 0.15 ± 0.05, τ = 16.5 ± 3.2 min. 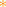 and 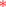, *p* < 0.05 for N33T and R56Q, respectively, compared to WT; two-way ANOVA followed by Dunnett’s test.

The discrepancy between the biochemical and flow cytometry approaches in the assignment of WT-like properties to N33T, R56Q, and A57P channels was interesting as it suggested that the presence of a WT-like abundance of the 155-kDa mature protein does not necessarily translate to normal channel surface density. On the basis of the flow cytometry approach, these mutants would be more appropriately characterized as temperature correctable, rather than WT-like (**Figures 4A–D**).

It was instructive to consider the potential mechanisms contributing to the deficits in surface expression of these mutant channels despite their WT glycosylation patterns. In principle, these could be mediated by impaired forward trafficking, decreased stability of the channel at the surface, or a combination of both mechanisms. An advantage of the flow cytometry approach is it can be adapted to probe these possibilities utilizing two complementary, optical pulse-chase assays (Kanner et al., 2017). To test differences in forward trafficking, we utilized a live-cell assay to analyze the delivery of new channels to the surface over time (**Figure 4E**). Live, non-permeabilized cells expressing WT or mutant BBS-hERG-YFP channels at 37°C for 24 h were moved to 4°C to halt all trafficking processes and subsequently exposed to unconjugated BTX to block all extracellular BBS epitopes initially present at the plasma membrane (pulse). Cells were then incubated at 37°C for varying time periods (chase), after which cells were returned to 4°C and the newly delivered surface channels labeled with BTX_647_ and quantified by flow cytometry. Control cells expressing WT BBS-hERG-YFP demonstrated a steady delivery of new channels to the surface (**Figure 4F**). In contrast, all three mutants demonstrated impaired delivery of new channels to the surface as evident by a significantly reduced plateau in BTX_647_ fluorescence (**Figure 4F**).

To determine the residence time of channels at the cell surface, we utilized a second optical, pulse-chase assay to measure removal of channels from the plasma membrane (**Figure 4G**). Live, non-permeabilized cells expressing WT and mutant BBS-hERG-YFP channels incubated at 27°C for 24 h (to ensure a comparable number of channels initially at the cell surface) were moved to 4°C and labeled with biotinylated bungarotoxin (BTX-biotin) at 4°C (pulse). Cells were then incubated at 37°C for varying time periods to resume trafficking (chase), and subsequently labeled with streptavidin-conjugated Alexa Fluor 647 (SA-647) at 4°C. In this paradigm, SA-647 labeling would only occur on channels that were initially present at the surface and labeled with BTX-biotin during the pulse period. As expected, WT hERG channels demonstrated an exponential decrease in surface labeling over time (**Figure 4H**). Interestingly, the three mutants demonstrated different rates of decline in surface labeling (**Figure 4H**), implying different rates of internalization, with R56Q displaying the most rapid removal from the cell surface.

## Discussion

In this study, we sought to develop a high-throughput optical flow cytometric assay that enables quantitative assessment of WT and LQT2 hERG channel trafficking, and rescue of trafficking-deficient mutants by low temperature or pharmacological chaperones. The method is complementary to other previously described approaches to monitor hERG channel trafficking, including a biochemical assay that assesses relative abundance of immature 135-kDa and fully glycosylated 155-kDa forms of the protein. By comparing results from the flow cytometric analyses of 16 PAS domain mutant hERG channels to published data of the same mutations assessed by the biochemical approach, we find not only areas of concordance that validates the assay, but also some discrepancies that highlight advantages of the flow cytometry method. We discuss the flow cytometry assay and our results in the context of established methods to monitor hERG channel trafficking and some of the results obtained with these approaches.

### Complex Glycosylation as a Marker for hERG Maturation and Surface Trafficking

The first studies elucidating glycosylation as a critical player in the maturation of WT hERG channels were reported almost two decades ago (Zhou et al., 1998b, 1999; Petrecca et al., 1999). It was further shown that while certain LQT2 mutations displayed post-translational processing similar to WT, others exhibited an impaired maturation evident as an absence of the 155-kDa fully processed protein band (Zhou et al., 1998a; Anderson et al., 2006, 2014). Moreover, treatment of cells with protease led to the digestion and disappearance of the mature 155-kDa band, with no effect on the immature 135-kDa band, consistent with the mature protein being predominantly at the cell surface (Zhou et al., 1998b; Rajamani et al., 2006). Consequently, biochemical analyses of the relative abundance of 135- and 155-kDa hERG bands has been a standard widely adopted tool to analyze trafficking of WT and mutant hERG channels under different conditions. Nevertheless, it is noteworthy that Gong et al. (2002) demonstrated that while N-linked glycosylation of hERG occurs at residue N598, mutating this site did not abolish hERG channel surface expression despite the disappearance of the 155-kDa form of the protein. Hence, while glycosylation is important for hERG maturation, it is not absolutely required for assembly and expression of functional hERG channels at the cell surface. This suggests the possibility that a sole reliance on the biochemical assay to categorize mechanisms underlying LQT2 mutations could potentially misclassify some as being in the Class 2 trafficking-deficient category.

There has been relatively scant research on the prevalence of the opposite phenomenon: Does a WT-like glycosylation pattern guarantee channel surface density similar to WT hERG? Previous studies found that despite rescue of the mature 155-kDa band in antidepressant-induced and inherited G601S LQT2 by lysosomal inhibitors and microtubule depolymerization, respectively, there was no subsequent rescue in functional hERG expression at the surface membrane (Dennis et al., 2011; Smith et al., 2011). Of the 16 PAS domain LQT2 mutations we studied, three had been previously classified as WT-like on the basis of their glycosylation patterns (Anderson et al., 2014). Remarkably, we found that all three exhibited significantly reduced surface expression compared to WT hERG, demonstrating lack of an absolute correlation between relative abundance of the 155-kDa mature protein form and its level of expression at the cell surface. We further found that the putative WT-like LQT2 mutant channels displayed impaired forward trafficking. Given that these mutants have WT glycosylation patterns, they have presumably reached or passed through the Golgi compartment. We cannot distinguish from our results whether the impaired forward trafficking arises from deficits in transport from the Golgi to the cell surface, or whether it mostly reflects shortfalls in some post-Golgi recycling compartments. This is pertinent as robust Rab11-mediated recycling of hERG channels has been observed (Lamothe and Zhang, 2013; Chen et al., 2015).

### Differential Plasma Membrane Stability of LQT2-Causing Mutants

Our flow cytometry method indicated that the residence time of WT hERG channels at the cell surface was short, with an apparent half-life of ∼9 min. The measured half-life was even shorter with the LQT2 mutations R56Q (∼5.7 min), and to a lesser extent N33T (7.3 min), but not A57P (∼11.7 min). Overall, the flow cytometry approach suggests a more rapid removal of surface hERG channels than has been previously reported. There have been several approaches applied to analyze the turnover or stability of hERG channels at the cell surface. One method uses the biochemical approach to follow the loss of the fully glycosylated hERG protein band in cells treated with brefeldin A. The half-life for the disappearance of the fully glycosylated hERG protein from this approach is on the order of ∼10 h. This biochemical method measures the turnover of mature hERG proteins, and is distinguished from our approach which directly measures the removal of surface channels. Notably, it has been demonstrated using the biochemical approach that some LQT2 mutations, including R56Q, display a decreased protein stability compared to WT hERG (Ke et al., 2013). Our finding that R56Q is more rapidly removed from the cell surface is in broad agreement with this previous report.

Another approach similar in principle to the method described here utilizes a hemagglutinin (HA) tag engineered into the extracellular S1-S2 loop of hERG. The labeling and fate of surface channels are then detected either by confocal microscopy in single cells, or cell surface ELISA in a population of cells (Wible et al., 2005; Apaja et al., 2013; Karnik et al., 2013). Apaja et al. (2013) utilized the cell surface ELISA approach to measure the plasma membrane residence time of WT and LQT2 hERG proteins. They measured a plasma membrane half-life of the WT protein of ∼ 9 and 3 h in HeLa and H9C2i cells, respectively. It is not clear why the two methods, which seem similar in principle, give rise to such disparate values for the residence time of hERG channels in the plasma membrane. One possibility is that the different cell types used in the studies could have an impact. To this point, Apaja et al. (2013) observe a threefold difference in the hERG plasma membrane residence time between HeLa and H9C2i cells. Another factor could be the stable versus transient expression of hERG channels in heterologous systems. As demonstrated in several studies with cystic fibrosis transmembrane conductance regulator (CFTR) trafficking, differences among absolute half-lives of channels at the cell surface may appear in heterologous systems and primary cells (albeit with similar relative changes of mutant relative to WT) (Sharma et al., 2004; Swiatecka-Urban et al., 2005; Cholon et al., 2010). Thus, future studies applying the BTX_647_ labeling method in the native cellular context of primary adult rodent cardiomyocytes will be important for distinguishing among these different possibilities.

A caveat for approaches that utilize epitope and fluorescent protein tags is the potential for unanticipated effects on hERG channel trafficking. This concern is mitigated by our findings that: (1) the tagged WT hERG trafficks robustly to the cell surface, and (2) the impact of PAS domain mutations on hERG surface density is largely consistent with expectations based on previous analyses of glycosylation patterns and low temperature rescue.

### Flow Cytometry as a Versatile Assay to Classify Trafficking-Deficient hERG Mutants and Elucidate New Therapeutic Strategies

Beyond the enhanced sensitivity and capacity to increase mechanistic insights, an important advantage of the flow cytometry method is its versatility compared to existing approaches. Existing ELISA-based assays rely on total fluorescence/chemiluminescence from cell populations. The lack of single cell resolution limits comparisons to homogeneous populations and potentially overlooks critical points in quality control (i.e., punctate/apoptotic cells, variable protein expression). Confocal studies allow for cellular/subcellular visualization, but do not provide the ability to easily quantify a large number of cells in an unbiased fashion. The flow cytometry method combines benefits of both approaches, enabling rapid analyses of a large population of cells with single-cell resolution, accounting for variations in transfection efficiency, and permitting normalization of channel surface expression to total protein expression levels. As such, there is no need to make stable cell lines for different mutations, and the capability to simultaneously analyze many colors at a time allows for potential applications that require multiplexing. Lastly, recent development and availability of 96-well flow cytometry protocols allow this approach to be adapted for medium- to high-throughput formats to identify novel correctors of hERG channel trafficking in a mutation-specific manner (Krutzik and Nolan, 2006; Duensing and Watson, 2018).

Although we have not conducted functional studies on these LQT2 mutants, it is important to note that previous studies observed changes in gating kinetics in certain PAS domain mutations (Chen et al., 1999; Gianulis and Trudeau, 2011). As such, the mere rescue of trafficking deficiencies may not be sufficient for therapeutic rescue of hERG function in the complex electrical milieu of the cardiac action potential (Perry et al., 2016). This highlights the need for combinatorial approaches for treating LQT2 pathology at both the cell biological (i.e., impaired forward trafficking, reduced residency time at the cell surface), and biophysical (i.e. conductance, gating kinetics) level. As there is no single mutation that is dominant in LQTS (with more variants being continually discovered), the use of new methods, such as flow cytometry, to further hone existing classifications of mutations and elucidate therapeutic subclasses will be critical in the pursuit of precision medicine for inherited arrhythmias and other ion channelopathies.

## Materials and Methods

### Molecular Biology and Cloning of Plasmid Vectors

The BBS-hERG-YFP constructs were engineered on the previously described hERG1a-YFP template (Puckerin et al., 2016), which utilized overlap extension PCR to fuse enhanced yellow fluorescent protein (EYFP) in frame to the C-terminus of hERG1a. A 13-residue bungarotoxin-binding site (BBS; TGGCGGTACTACGAGAGCAGCCTGGAGCCCTACCCCGAC) (Sekine-Aizawa and Huganir, 2004; Yang et al., 2010) was then introduced between residues T436/E437 in the extracellular S1–S2 loop of hERG using the Quik-Change Lightning Site-Directed Mutagenesis Kit (Stratagene) according to the manufacturer’s instructions. 16 LQT2 mutations were introduced in the PAS domain of BBS-hERG-YFP via site-directed mutagenesis.

### Cell Culture and Transfections

Low passage human embryonic kidney (HEK293) cells were cultured at 37°C in Dulbecco’s Modified Eagle’s Medium (DMEM) supplemented with 8% fetal bovine serum (FBS) and 100 mg/mL of penicillin–streptomycin. HEK293 cell transfection was accomplished using the calcium phosphate precipitation method. Briefly, plasmid DNA was mixed with 7.75 μL of 2M CaCl_2_ and sterile deionized water (to a final volume of 62.5 μL). The mixture was added dropwise, with constant tapping to 62.5 μL of 2x Hepes buffered saline containing (in mM): HEPES 50, NaCl 280, Na_2_HPO_4_ 1.5, pH 7.09. The resulting DNA–calcium phosphate mixture was incubated for 20 min at room temperature and then added dropwise to HEK293 cells (60–80% confluent). Cells were washed with Ca^2+^-free phosphate buffered saline after 4–6 h and maintained in supplemented DMEM.

### Flow Cytometry Assay of Total and Surface Q1 Channels

Cell surface and total ion channel pools were assayed by flow cytometry in live, transfected HEK293 cells as previously described (Yang et al., 2010; Aromolaran et al., 2014). Briefly, 48 h post-transfection, cells cultured in 12-well plates gently washed with ice cold PBS containing Ca^2+^ and Mg^2+^ (in mM: 0.9 CaCl_2_, 0.49 MgCl_2_, pH 7.4), and then incubated for 30 min in blocking medium (DMEM with 3% BSA) at 4°C. HEK293 cells were then incubated with 1 μM Alexa Fluor 647 conjugated α-bungarotoxin (BTX-647; Life Technologies) in DMEM/3% BSA on a rocker at 4°C for 1 h, followed by washing three times with PBS (containing Ca^2+^ and Mg^2+^). Cells were gently harvested in Ca^2+^-free PBS, and assayed by flow cytometry using a BD LSRII Cell Analyzer (BD Biosciences, San Jose, CA, United States). CFP- and YFP-tagged proteins were excited at 407 and 488 nm, respectively, and Alexa Fluor 647 was excited at 633 nm.

Optical pulse chase assays to monitor rates of channel forward trafficking and internalization were conducted on live, transfected HEK293 cells as previously described (Kanner et al., 2017). For the forward trafficking studies, cells were incubated at 37°C for 24 h prior to the experiments. Cells were placed on 4°C to halt trafficking processes and washed twice with PBS containing Ca^2+^ and Mg^2+^. For forward trafficking experiments, cells were incubated with 3 μM untagged BTX in DMEM/3% BSA at 4°C for 1 h to block surface channels, and then washed three times with PBS containing Ca^2+^ and Mg^2+^. Cells were incubated with DMEM/3% BSA and placed at 37°C to resume trafficking for different time intervals (0, 10, 20, 40, 60 min). Cells were then returned to 4°C and newly delivered channels were labeled with 1 μM BTX-647 in DMEM/3% BSA for 1 h. Finally, cells were washed three times with PBS containing Ca^2+^ and Mg^2+^, gently harvested in Ca^2+^-free PBS, and assayed by flow cytometry. For surface stability/internalization experiments, cells were incubated at 27°C for 24 h prior to the experiments. Cells were placed on ice (4°C) to halt trafficking processes and washed twice with PBS containing Ca^2+^ and Mg^2+^. Cells were then incubated in DMEM/3% BSA blocking medium for 30 min at 4°C followed by a 1 h incubation at 4°C (pulse) with 1 μM biotinylated α-bungarotoxin (BTX-biotin; Life Technologies), with gentle rocking. Cells were washed three times in PBS containing Ca^2+^ and Mg^2+^ and placed in DMEM/3% BSA at 37°C for different time intervals (0, 10, 20, 40, 60 min) to resume trafficking (chase). Cells were returned to 4°C, washed once with PBS, and channels remaining at the surface were labeled with streptavidin-conjugated Alexa Fluor 647 (Life Technologies). Finally, cells were washed three more times with PBS with Ca^2+^ and Mg^2+^, harvested in Ca^2+^-free PBS, and assayed by flow cytometry.

### Data and Statistical Analyses

Data were analyzed off-line using FlowJo, Microsoft Excel, Origin and GraphPad Prism software. Statistical analyses were performed in Origin or GraphPad Prism using built-in functions. Statistically significant differences between means (*p* < 0.05) were determined using two-way ANOVA, followed by Dunnett’s correction for multiple comparisons. Data are presented as means ± SD.

## Author Contributions

SK designed and conducted the experiments, analyzed and interpreted the data, and wrote the manuscript. AJ conducted the experiments and analyzed the data. HC designed the experiments, analyzed and interpreted the data, and wrote the manuscript.

## Conflict of Interest Statement

The authors declare that the research was conducted in the absence of any commercial or financial relationships that could be construed as a potential conflict of interest.
